# The carbon footprint of Italian schools meals: An optimal choice of dishes in vegan, vegetarian, and omnivorous menus

**DOI:** 10.3389/fnut.2022.854049

**Published:** 2022-08-31

**Authors:** Luca Benvenuti, Alberto De Santis, Marika Ferrari, Deborah Martone, Laura Rossi

**Affiliations:** ^1^Department of Computer, Control and Management Engineering, Sapienza University of Rome, Rome, Italy; ^2^CREA Council for Agricultural Research and Economics, Research Centre for Food and Nutrition, Rome, Italy

**Keywords:** binary linear programming (BLP), carbon footprint (CF), school menus, vegan diet, vegetarian diet

## Abstract

This study aims to assess the carbon footprint associated with vegan, vegetarian, and omnivorous menus for primary school lunches in Italy. For this purpose, healthy and acceptable menus with minimal greenhouse gas emissions have been designed by a binary linear programming model. The results show that the adoption of a specific diet may help in reducing the carbon footprint of menus, but it is the optimal selection of dishes that ultimately makes the difference. Interestingly enough, the optimal choice of dishes and the restriction of meat consumption in omnivorous menus can lead up to a 40% emission reduction compared to the current school lunch menu of the municipality of Rome. Moreover, the optimal choice of dishes in vegan menus provides the menu with the lowest carbon footprint among all kinds of diets.

## Highlights

- The optimal selection of dishes in menu design is the main driver to reducing GHGE.
- A generic vegan (vegetarian) menu may impact more than a vegetarian (omnivorous) menu.
- The optimal selection of dishes in vegan menus provides the lowest impact.
- The optimal selection of dishes in omnivorous menus leads to a 40% GHGE reduction compared to actual menus.

## Introduction

The food system is a primary sector of the economy that accounts for 34% of total anthropogenic GHG emissions (1). Hence, reducing the related GHG emissions is at the heart of the European Green Deal (2) that, through the Farm to Fork Strategy (3), aims to make food systems fair, healthy, and environmentally friendly. In more detail, one of the goals is to ensure food security, nutrition, and public health, making sure that everyone has access to sufficient, safe, nutritious, and sustainable food. This corresponds to the promotion of sustainable diets as defined by the Food and Agriculture Organization of the United Nations (4), that is “diets with low environmental impacts which contribute to food and nutrition security and healthy life for present and future generations. Sustainable diets are protective and respectful of biodiversity and ecosystems, culturally acceptable, accessible, economically fair and affordable; nutritionally adequate, safe and healthy; while optimizing natural and human resources.”

Adopting a sustainable diet can effectively help to reach the goal since diets and consumer behaviors strongly affect the food system. In fact, because of the relationship between supply and demand, the choices of consumers drive what, and how much, is produced. For example, the choice of locally sourced and organic food helps reduce food miles and the use of pesticides, fertilizers, and antibiotics. In general, consistent evidence indicates that a dietary pattern higher in plant-based foods (e.g., vegetables, fruits, legumes, seeds, nuts, whole grains) and lower in animal-based foods (especially red meat), as well as lower in total energy, is both healthier and associated with a lesser impact on the environment (5–7). For example, the replacement of a meat meal with a vegan or a vegetarian meal significantly helps to lower GHG emissions (8).

Diets and consumer behaviors, that is food preference and preparation techniques, are greatly affected by traditions, beliefs, and values shared by a community. They define the structure of each meal and the set of foods and dishes that are appreciated and considered edible and acceptable (9). Cultural habits are quite rooted in the population and therefore a shift in consumer behaviors may require some effort. In this respect, school is a privileged environment for acquiring and solidifying eating habits through health and nutrition education as well as environmental awareness.

School lunch programs have contributed significantly to shaping dietary habits (10, 11) in line with dietary recommendations. Indeed, school meal menus are classically designed to promote healthy eating habits aimed to prevent overweight and obesity and their consequences in terms of the incidence of non-communicable diseases (12). Italian National guidelines for school feeding programs are provided by the Ministry of Health (13). These guidelines address nutritional issues and also introduce special therapeutic diets, for example for food intolerance and allergies. Moreover, intercultural menus are promoted to accommodate the eating habits of an increasing foreign school population, and special menus are guaranteed for ethical, religious, and cultural reasons. Therefore, vegan and vegetarian menus, as well as menus with religious restrictions such as pork-free recipes, can be requested. Further indications about the acceptability of the menus, and consequently the reduction of food waste, were released in 2018 (14).

The possibility of choosing either vegan or vegetarian menus, as well as the concern about food waste reduction, are the only items in the guidelines that, though indirectly, pursue the goal of reducing GHG emissions. This goal can be indeed further pursued by taking explicitly into account the GHG emissions in the design of school menus.

To this end, a suitable design tool has been recently developed (a detailed description can be found in (15)). The tool makes use of a binary (0–1 integer) programming method to provide the sequence and composition of daily lunches that, over a given period, minimize the GHG emissions needed for their preparation. The meals composing the lunches are chosen using dishes out of a given recipe book. Therefore, instead of defining just a level of consumption of food groups or food items, the tool provides a realistic menu. To cope with the nutritional recommendations, each dish is characterized by its energy and nutritional content. Furthermore, the variety of the menu can be explicitly taken into account in a very natural way, that is bounding the daily, weekly, or total repetitions of single dishes and dishes in the same food group. This is a basic issue to make the menus *acceptable*. Acceptability is however a broader concept implying the food preferences of the pupils. This is implicitly included in the optimization model since only the actual recipes from school menus are considered.

The tool has been used to design school lunch menus with minimal carbon or water footprint. In more detail, in (16) such a design was performed using the recipe book of the Municipality of Rome, while a sample of 52 Italian school menus from Italian macro-regions (North, South, Center, and Islands) was used in (17). The results show that a significant reduction of the environmental impact can be obtained while satisfying all the nutritional and acceptability recommendations.

This study aims to use the above tool to compare the GHG emissions associated with different kinds of menus, such as vegan, lacto-ovo vegetarian, and omnivorous, for a two-week schedule for the primary school lunch. To this end, an extension of the recipe book in (17), including vegan recipes, was considered. These recipes are not included in the standard school lunch menus but are provided only by demand.

##  Materials and methods

The data consist of a set of dishes, whose composition and serving sizes are fixed, for which the energy, nutrient contents, and carbon footprint are available. The schedule must be organized by choosing within the given set of dishes the sequence of daily lunches while satisfying some constraints related to adequate energy and nutrient intakes and food acceptability. These constraints are related to the composition of the lunch (first and second-course dish, side dish, bread, and fruit), daily and weekly bounds on energy and nutrient intakes, and bounds on weekly and total repetitions of dishes in the same food group.

### Recipe book, nutritional and carbon footprint data of dishes

A sample of 52 Italian primary school menus (children aged 6–11 years) was collected, by considering all the Italian macro-regions (North, South, Center, and Islands). From the menus, single dishes were extracted to have a list of first-course dishes (in general including pasta or other carbohydrate sources), a list of second-course dishes (in general a source of protein), a list of side dishes (vegetables, potatoes, or salad), fresh fruit, and bread. Each dish has a fixed portion size, according to the Italian recommended dietary allowances (RDAs) (18). The final sample consists of 209 dishes grouped as 69 first–course, 103 second–course, 35 side dishes, fruit^1^, and bread. Tap water is the only beverage allowed for lunch. The sample of dishes collected seems to cover all the typical recipes in Italian school meals since checking some further menus did not provide new dishes significantly differing from the already collected ones. It includes vegan, vegetarian, and omnivorous dishes as reported in Table 1.

**Table 1:** Number of first-course, second-course, and side dishes divided according to the ingredients food group.

	Vegan recipes*	**Eggs, dairy recipes**	**Vegetarian recipes^§^**	**Red meat recipes**	**White meat recipes**	**Fish recipes**	**Omnivorous recipes^§^**
First-course dishes	36	16	52	11	0	6	69
Second-course dishes	21	14	35	33	12	23	103
Side dishes	25	9	34	1	0	0	35

*Some first course dishes are not vegan just because they are topped with a sprinkling of parmesan cheese. When designing a vegan menu this topping was not considered in the recipe.

^§^Vegetarian recipes include vegan recipes and eggs and dairy recipes; Omnivorous recipes include vegetarian recipes and recipes with meat and fish.

The energy and nutrient composition of the dishes were calculated from their ingredients using the Italian Food composition and Nutrition database (19) completed with the French Food composition and Nutrition database in case of missing items (20). The GHG emitted to prepare each dish is computed from its ingredients using the database developed in the framework of the EU SU-EATABLE LIFE project (21). The missing items, mainly corresponding to vegan ingredients, were retrieved from the Supplementary material given in (22). The list of dishes considered and the corresponding energy and nutrient content, and carbon footprint, are given in Supplementary Tables SM1–SM3.

The bounds on energy and nutrient intakes were established based on Italian recommendations (18), and their average values for lunch in primary school canteens were obtained considering that this meal should cover 35% of the daily amount as recommended by Italian Guidelines for healthy eating (23). Energy and nutrient intakes variability is guaranteed by defining appropriate daily and weekly ranges around average values, as reported below in Table 2^2^. The ranges are chosen to allow a slightly larger variation on the single lunch while keeping the weekly intake closer to the average. This corresponds to focusing on nutritional adequacy mainly on a weekly basis.

**Table 2:** Lunch energy and nutrient constraints for children aged 6–11 years.

	**Daily average value**	**Daily range**		**Weekly average value**	**Weekly range**	
		**Lower**	**Upper**		**Lower**	**Upper**
Energy (kcal)	700	500	900	3,500	3,000	4,000
Carbohydrates (g)	100	70	130	500	450	550
Protein (g)	27.5	15	40	137.5	100	175
Fat (g)	25	10	40	125	100	150
Sugar (g)	20	0	40	100	50	150
Fiber (g)	10	0	20	50	25	75
Sodium (mg)	400	100	700	2,000	1,500	2,500
Calcium (mg)		-	-	-	200 ×5	-
Iron (mg)		-	-	-	5 ×5	-
Vitamin B_12_ (μg)		0.35	-	-	-	-

Constraints were imposed also for fiber, sodium, calcium, and iron, in addition to those fixed for macronutrients. Fiber consumption is particularly low in Italy in the considered age group (24), (25), thus it is important to stress adherence to the recommendation in menu design. On the other hand, restriction on sodium intake was set up considering the preventive value of early reduction of salt intake in this age group, as recommended by WHO (26). A weekly lower bound for iron and calcium intake was considered based on the age-specific dietary allowance recommendation (18). Similarly, a daily lower bound for vitamin B_12_ was also considered according to (18). The bounds on lunch intake of calcium and vitamin B_12_ do not represent 35% of recommended intake. Indeed, they are determined by taking into account that, in a non-vegan diet, Italian breakfasts include a glass of milk and then provide a significant amount of these two nutrients (27). As a matter of fact, a breakfast glass of milk (200 ml) contains on average 246 mg of calcium and 1 μg of vitamin B_12_^3^.

Some further recommendations consist in avoiding eating processed meat due to public health authorities' recommendations (28). The meal plan covers the school lunches over 2 weeks. Variety of the menus is accomplished by avoiding that the same dish appears twice in the meal plan. Moreover, the total number of dishes in the same food group is constrained within a defined range to design menus with different frequencies of red meat, dairy products, etc …. In addition, this allows characterizing omnivorous, vegetarian, and vegan menus.

### Mathematical modeling and optimization method

The problem consists of specifying, for every day *d* (*d* = 1, ⋯ , 5) and every week *w* (*w* = 1, 2), the set of dishes composing the lunch. To this end, a binary variable $x_{d,w}^{i}$ is associated with every recipe *i* (*i* = 1, ⋯ , 209): it assumes value 1 if the *i*-th dish is part of the lunch of the day *d* of the week *w*, and 0 otherwise. Therefore, the quantity $Q_{d,w}^{k}$of the *k*-th item (energy, fat, …, CO_2_ equivalent) in the lunch of the day *d* of the week *w* is:


$$\begin{array}{l} Q_{d,w}^{k}=\overset{209}{\underset{i=1}{\sum }}x_{d,w}^{i}\times Q_{i}^{k}\; \end{array}$$

where $Q_{i}^{k}$ is the *k*-th item content in the *i*-th dish, as reported in the Supplementary material. The quantity $Q_{w}^{k}$ of the *k*-th item in the week *w* and the quantity *Q*^*k*^ in the whole menu are:


$$\begin{array}{l} Q_{w}^{k}=\overset{5}{\underset{d=1}{\sum }}\;Q_{d,w}^{k},\;Q^{k}=\overset{2}{\underset{w=1}{\sum }}Q_{w}^{k}\; \end{array}$$

As previously discussed, quantities $Q_{d,w}^{k}$, and $Q_{w}^{k}$ are bounded by lower and upper limits, see Table 2, and this restricts the admissible schedule of dishes.

In addition to the nutritional requirements, the schedule must be healthy and varied. Variety is achieved by imposing that every dish can be proposed at most once on the menu and by limiting the total number of dishes of the same food group in the week and on the whole menu. The healthiness of the menu mainly depends on nutritional recommendations and is further pursued by avoiding the consumption of processed meat. The number of times that dishes in food group *G* (red meat, dairy, …) are proposed within the week *w* and in the menu are:


$$\begin{array}{l} R_{w}^{G}=\underset{i\in G}{\sum }\overset{5}{\underset{d=1}{\sum }}x_{d,w}^{i}\;,\;R^{G}=\overset{2}{\underset{w=1}{\sum }}R_{w}^{G}\; \end{array}$$

so that variety can be ensured by fixing lower and upper values for $R_{w}^{G}$ and *R*^*G*^. The menu is determined by minimizing the objective $Q^{CO_{2eq}}$, that is the total amount of CO_2eq_ emission. The objective is a linear function of the 2090 binary variables $x_{d,w}^{i}$ as follows:


$$\begin{array}{l} Q^{CO_{2eq}}=\overset{2}{\underset{w=1}{\sum }}\overset{5}{\underset{d=1}{\sum }}\overset{209}{\underset{i=1}{\sum }}x_{d,w}^{i}\times Q_{i}^{CO_{2eq}}\; \end{array}$$

where $Q_{i}^{CO_{2eq}}$ is the GHGE amount needed to serve the dish *i*.

Note that the problem is not a classical Linear Programming optimization problem since the variables may assume only two values (0 or 1) and do not vary continuously in a range. Therefore, the problem is a constrained binary linear programming one. The optimization was carried out by using the online version of CPLEX for AMPL on the NEOS server (https://neos-server.org/neos/); the model is extensively described in (15).

The optimization procedure provides a menu, that is a set of dishes and their daily schedule. From the mathematical point of view, the solution is not unique since, for example, different optimal menus can be obtained by a simple permutation of lunches within the days of the same week^4^. The set of dishes composing the menu may not be unique as well since, for example, there may be dishes sharing the same energy, nutrients, and environmental impact values that can be interchangeably used^5^. Furthermore, there may also be interchangeable combinations of dishes that, if present, could be identified only by an exhaustive enumeration of all admissible menus, however unpractical given their very huge number. The procedure provides just one of the optimal menus that may be considered as equivalent since they have the same carbon footprint and satisfy the nutritional constraints of Table 2, the acceptability constraints on lunch structure and dish repetitions, and the health recommendation on avoiding processed meat. However, several different optimal menus can be obtained by substituting interchangeable dishes or by a simple permutation of lunches within the days of the same week. This may allow obtaining a more palatable sequence of lunches.

The optimization procedure was applied to design four different two-weeks menus with minimal total GHGE. The first menu refers to a vegan diet and, according to Table 1, is obtained by selecting dishes among 36 first-course, 21 second-course, and 25 side dishes. The second menu is a vegetarian one and, according to Table 1, is obtained by selecting dishes among 52 first-course, 35 second-course, and 34 side dishes. The last two menus are omnivorous. Health (29) and environment (6) issues ask for a reduction in the consumption of animal-based food, especially red meat. Indeed, red meat produces the most greenhouse gas emissions and is related to a high risk of non-communicable chronic diseases, such as some cancers, cardiovascular and cerebrovascular diseases, and an overall impact on populations' mortality rates. To this end, one menu drastically agrees with the recommendation by totally avoiding red meat dishes, while the other includes a limited consumption of red meat. Hence, according to Table 1, the first omnivorous menu is obtained by selecting dishes among 58 first-course, 70 second-course, and 34 side dishes, the second one considers instead all the dishes in the recipe book.

## Results and discussion

It was not possible to design a vegan menu satisfying all the nutritional constraints of Table 2. This is due to the lack of vitamin B_12_ content in most vegan dishes since no fortified foods are considered. This is a well-known problem in vegan diets, usually solved by either considering fortified foods or by taking supplements. The vegan menu is then designed without constraints on the vitamin B_12_ intake and assuming the use of appropriate supplements for it and the breakfast intake of calcium. Moreover, to promote vegetable consumption, vegetable side dishes must be offered a minimum of four times per week. The optimal menu is given in Supplementary Table SM4 and has an average weekly GHGE equal to 1,742 g of CO_2eq_.

The vegetarian menu satisfies all the constraints given in Table 2, including that on vitamin B_12_ intake. In addition to the vegetable dishes frequency of the previous menu, both dairy and egg dishes must be proposed at least once a week but no more than twice a week and three times on the whole menu. The optimal menu is given in Supplementary Table SM5 and has an average weekly GHGE equal to 2,570 g of CO_2eq_.

In addition to the frequencies already fixed for vegetables, eggs, and dairy dishes, the first omnivorous menu must not contain red meat dishes, while white meat dishes must be proposed at least once a week but no more than twice a week and three times in the whole menu. The optimal menu is given in Supplementary Table SM6 and has an average weekly GHGE equal to 2,678 g of CO_2eq_.

The second omnivorous menu must contain, in addition to the food groups frequencies of the previous menu, a red meat dish once a week and a beef dish once in the whole menu^6^. The optimal menu is given in Supplementary Table SM7 and has an average weekly GHGE equal to 3,578 g of CO_2eq_.

**Figure 1:**
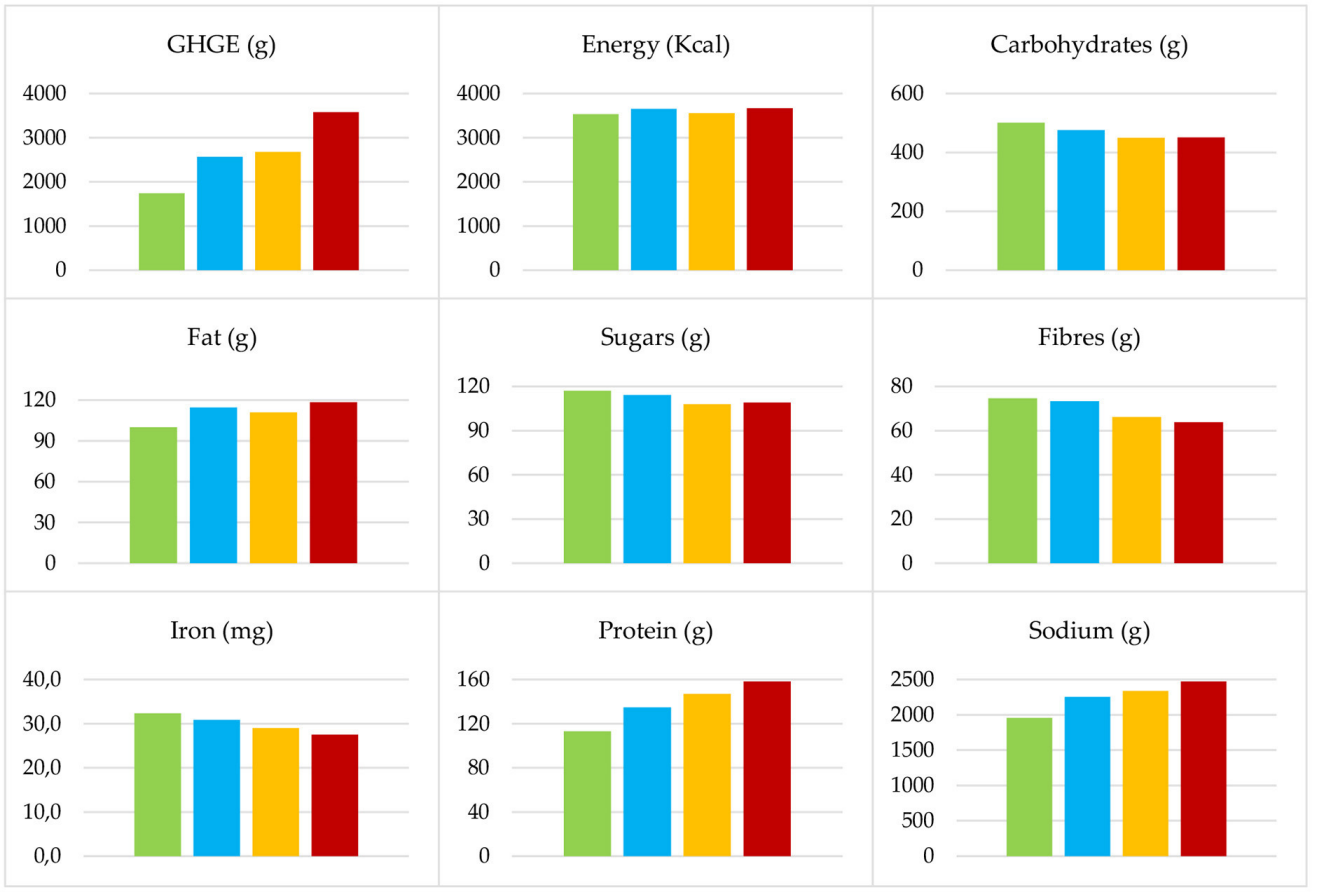
Weekly average energy, nutrients, minerals, and GHGE for: Vegan optimal menu 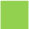, vegetarian optimal menu 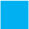, and first and second omnivorous optimal menus (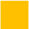 and 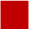, respectively).

A comparison of the nutritional content and carbon footprint for the above optimal menus is given in Table 3 and illustrated in Figure 1. Vitamin B_12_ has a daily lower bound of 0.35 μg and hence the value for the weekly lower bound in Table 3 is just five times the daily one and, as previously discussed, is not satisfied by the vegan menu.

**Table 3:** Weekly average energy, nutrients, minerals, and GHGE for the four optimal menus considered.

**Menu**	**Energy (Kcal)**	**Carbs (g)**	**Protein (g)**	**Fat (g)**	**Sugar (g)**	**Fiber (g)**	**Sodium (mg)**	**Calcium (mg)**	**Iron (mg)**	**Vit B_12_ (μg)**	**CO_2eq_ (g)**
Weekly lower bound	3,000	450	100	100	50	25	1,500	1,000	25	1.75	N/D
Weekly upper bound	4,000	550	175	150	150	75	2,500	N/D	N/D	N/D	N/D
Vegan	3,533	501	113	100	117	75	1,956	1,003	32.3	0.01	1,742
Vegetarian	3,654	476	135	115	114	73	2,255	1,509	30.8	2.89	2,570
Omnivorous (1)	3,555	450	147	111	108	66	2,340	1,292	29.0	3.80	2,678
Omnivorous (2)	3,665	451	158	119	109	64	2,472	1,265	27.5	3.67	3,578

**Figure 2:**
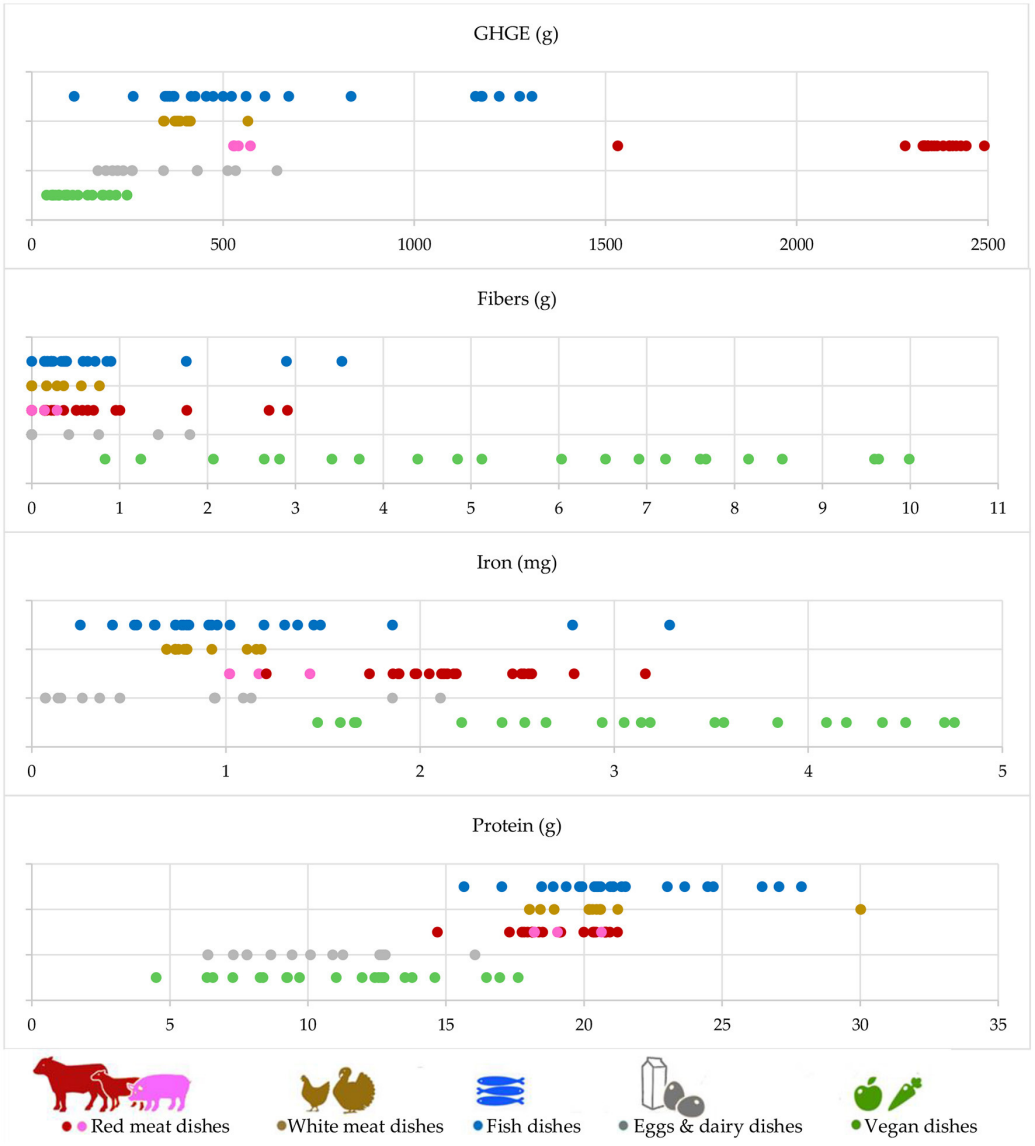
GHGE (grams of CO_2eq_) and some nutrients and minerals associated with the second-course dishes considered in the recipe book (excluding dishes with processed meat). Eggs and dairy dishes can be offered on the vegetarian and omnivorous menus, meat, and fish dishes can be offered only on the omnivorous menus.

As Figure 1 makes clear, the GHGE doubles from the vegan optimal menu to the omnivorous optimal menu of the second kind. This variation mostly depends on the second-course dishes, since all the menus share similar first-course and side dishes. The carbon footprint significantly increases when passing from the vegan optimal menu to the vegetarian optimal menu and from the omnivorous optimal menu of the first kind to that of the second kind. The first GHGE variation (+48%) is due to the addition of eggs and dairy dishes to the diet; the second variation (+34%) is due to the addition of red meat, in particular beef meat. Interestingly enough, the GHGE related to the vegetarian optimal menu and the omnivorous optimal menu of the first kind is quite the same, because only fish and white meat dishes were added to the menu. This is made evident by the first picture in Figure 2, where the GHGEs associated with the second-course dishes are depicted. Indeed, eggs and dairy, fish, and white meat dishes show similar GHGE values. On the other hand, vegan dishes have the lowest impact while ruminant meat dishes have the highest one.

Let us now consider the nutritional characteristics of the four optimal menus. The weekly average values of energy, carbohydrates, fat, and sugars are quite the same. They show a deviation of a maximum of 10% from their average on the four menus. The other nutrients and minerals show more evident variations. In particular, as shown in Figure 1, fibers and iron decrease when passing from one menu to the other, with a total variation equal to −15% from the vegan optimal menu to the omnivorous optimal menu of the second kind. As above, this is also made evident by the second and third pictures in Figure 2 that show how vegan second-course dishes, consisting mainly of legumes, are richer in fiber and iron. On the contrary, as shown in Figure 1, the intake of protein and sodium increases when passing from one menu to the other, with a total variation equal to +40 and +26%, respectively. Again, the protein increase can be explained considering the protein content distribution over the dishes, given in the fourth picture in Figure 2.

Note that the average values for all the menus of energy and nutrients over the 2 weeks do not attain the maximum weekly allowed values (save for Fiber in the vegan menu). This means that the optimal menus do not provide maximum values for all the weeks. Should it happen and be an issue, two model improvements are possible to deal with it: either narrow the weekly ranges or add a further range on the whole menu.

As a general remark, we can observe that the vegan optimal menu has the lowest carbon footprint and that the introduction of just eggs and dairy in the diet significantly worsens the impact. Although the adoption of a vegan diet helps in reducing the carbon footprint of a menu, it is the optimal selection of dishes that ultimately makes the difference. Indeed, the GHGE associated with a generic vegan menu can be much greater than that associated with the vegan optimal menu. The maximum GHGE of a menu which is nutritionally adequate can be computed by just maximizing the total amount of CO_2eq_ emission instead of minimizing it. In the case of vegan menus, this results in a menu (see Supplementary Table SM8) with an average weekly GHGE equal to 2,937 g of CO_2eq_ which has an impact greater than the vegetarian optimal menu and the omnivorous optimal menu of the first kind, respectively (see Supplementary Tables SM5, SM6). Hence, eating vegan is not sufficient to guarantee a pressure reduction on the environment, but it is also necessary to make an appropriate selection of dishes.

Similar arguments hold when considering vegetarian menus. In particular, the most impacting vegetarian menu (see Supplementary Table SM9) produces an average weekly GHGE equal to 4,723 g of CO_2eq_ which is far higher than that produced by the omnivorous optimal menu of the second kind (see Supplementary Table SM7). In other words, a non-optimal choice of dishes in the design of a vegetarian menu may lead to an environmental pressure higher than that of some omnivorous menus.

**Figure 3:**
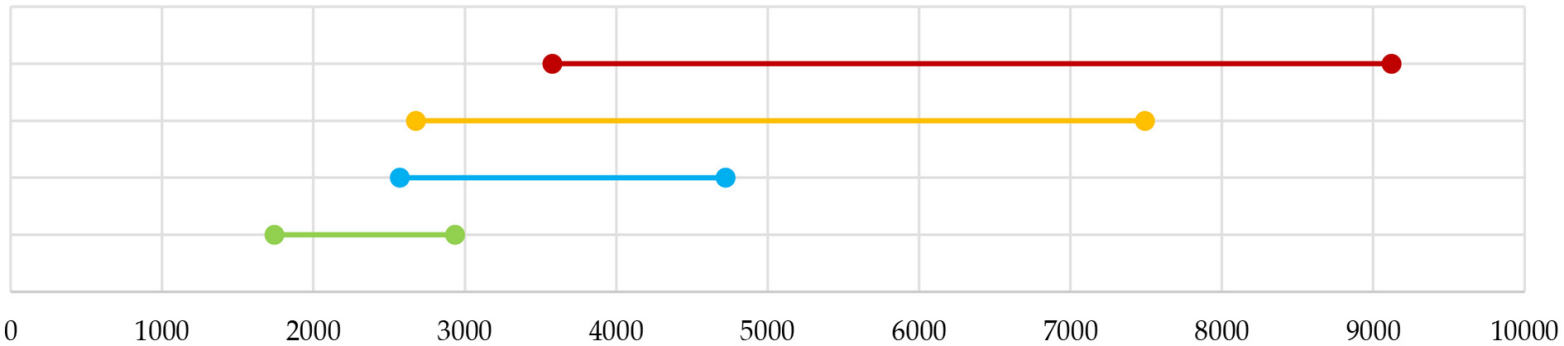
Weekly average GHGE ranges for vegan menus 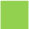, vegetarian menus 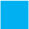, and first and second omnivorous menus (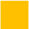 and 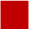, respectively). The upper values for the omnivorous menus are 7,493 and 9,123 g.

Figure 3 makes clear these results by showing that the possible values of GHGE associated with generic vegan, vegetarian, and omnivorous menus partially overlap. This means that there are vegan menus producing pressure on the environment higher than some vegetarian menus. Similarly, there are vegetarian menus that exhibit higher GHGE than some omnivorous menus.

In general, adding fish and non-ruminant meat to the vegetarian menu does not change its overall impact, but ruminant meat consumption determines a further worsening. The impact increment depends on the meat food groups introduced and on their frequencies on the menu. Nevertheless just optimizing the menu, i.e., the optimal selection of the dishes composing the menu may reduce significantly its carbon footprint. This is made evident by computing the carbon footprint of the current school lunch menu proposed by the municipality of Rome. This menu is defined over 9 weeks and has nutritional values in line with the daily and weekly bounds given in Table 2^7^. It has been developed by nutritionists and it is guaranteed to be nutritionally adequate and balanced in carbohydrates, fats, and proteins, along with all essential minerals, vitamins, and health-promoting substances. It is quite rich in every food group and can hence be compared with the omnivorous optimal menu of the second kind that contains exactly 1 second-course dish with fish, one with red meat, one with white meat, one with eggs, and one with dairy, every week. The weekly GHGE average of the menu of Rome is equal to 5,841 g of CO_2eq_ so the omnivorous optimal menu shows a reduction by about 40% of the carbon footprint. This is precisely in line with the target of the European Green Deal^8^ and is mainly due to the optimal selection of dishes made in the design of the menu. For example, the two red meat dishes on the menu are a dish with pork (roast pork) and a dish that contains beef together with pork (beef/pork meatballs). Indeed, the menu must contain one red meat dish once a week and one beef dish once in the whole menu, and roast pork and beef/pork meatballs are the dishes with minimal GHGE among all the red meat dishes and red meat dishes with beef, respectively.

The properties of the menus considered in this paper are similar to those at the single recipe level presented in (22), where a total of 311 recipes from six different German omnivorous, vegetarian and vegan cookery books were analyzed in terms of GHG emissions and costs. Moreover, the results are in line with the observational studies on the adult population made in France (8) (using a food frequency questionnaire over about 29,000 participants), the UK (32) (using a food frequency questionnaire over about 55,500 participants aged 20–79), and Italy (33) (using a 7-days dietary record over 153 participants), in which the environmental impacts of different diets with different levels of animal product consumption were compared. Similar results were obtained by designing diet plans using linear programming (34, 35). In particular, the diet plans are obtained by minimizing their deviation from a reference/current diet while fulfilling nutrient and climate footprint constraints. Constraining the diet GHG emissions results in a reduction in meat, dairy products, and processed foods.

In general (36), the results available in the literature show that diets and observed dietary patterns in the population are less impacting on the environment the higher they are rich in plant-based foods. In more detail, the results suggest that the vegan diet is the optimal diet for the environment because, out of all the other diets, its production results in the lowest level of GHG emissions. Nevertheless, there is the possibility of significantly reducing the environmental impact of a given diet, without excluding the meat and dairy food groups, but rather, by reducing them substantially.

Our findings confirm and enhance these results. Indeed, they show that the above properties of diets and observed dietary patterns remain true when considering school lunch menus with minimal GHGE. This conclusion is not obvious since the tool used in this work provides realistic menus in place of diet plans. Moreover, it is shown that although the adoption of a specific diet may help in reducing the carbon footprint, it is the optimal selection of dishes that ultimately reduces the impact of a menu.

## Conclusions

This study shows that, in general, a low carbon footprint menu is plant-based. It also results that meat consumption, and in particular ruminant meat, is the most important contributor to the carbon footprint within the protein source dishes. Hence, meat dishes should be partially replaced with legumes, cereals, fruits, and vegetables. Anyway, to comply with cultural habits, it is possible to include on the menu fish and white meat dishes that are nutritionally beneficial and more sustainable than red meat dishes.

The added value of the proposed methodology, based on an integer optimization approach, is that the choice of a particular diet (vegan, vegetarian) does not, *per se*, guarantee a GHGE reduction as compared to a generic menu: it is the optimal selection of dishes on the menu that determines the minimal carbon footprint. Moreover, the optimal choice of dishes on omnivorous menus can lead up to a 40% emission reduction compared to the current school lunch menu of the municipality of Rome. This allows to progressively shift food behavior toward the direction of environmental protection, avoiding drastic changes to achieve environmental objectives step by step.

This result could be further improved by considering aspects related to the promotion of food short supply chain and seasonal products, as well as agricultural and processing techniques (37).

## Data availability statement

The original contributions presented in the study are included in the article/Supplementary material, further inquiries can be directed to the corresponding author.

## Author contributions

The research questions, conceptualization, design of the study were carried out, data were analyzed, software was designed, and writing and original draft preparation was carried out by LB and AD. The methodology was revised and database compilation was carried out by MF, DM, and LR. Data interpretation was undertaken and writing, review, and editing were done by LR, MF, DM, LB, and AD. All authors have read and agreed to the published version of the manuscript.

## Funding

This paper was funded by SYSTEMIC An integrated approach to the challenge of sustainable food systems: adaptive and mitigatory strategies to address climate change and malnutrition, within the Knowledge Hub on Nutrition and Food Security, which has received funding from national research parties in Belgium (FWO), France (INRA), Germany (BLE), Italy (MIPAAF), Latvia (IZM), Norway (RCN), Portugal (FCT), and Spain (AEI) in a joint action of JPI HDHL, JPI-OCEANS, and FACCE-JPI launched in 2019 under the ERA-NET ERA-HDHL (No. 696295).

## Conflict of interest

The authors declare that the research was conducted in the absence of any commercial or financial relationships that could be construed as a potential conflict of interest.

## Publisher's note

All claims expressed in this article are solely those of the authors and do not necessarily represent those of their affiliated organizations, or those of the publisher, the editors and the reviewers. Any product that may be evaluated in this article, or claim that may be made by its manufacturer, is not guaranteed or endorsed by the publisher.

## Footnotes

^1^It is assumed that seasonal fruit is administered. Since the proposed menu does not refer to a specific season, then a generic fruit is considered with nutrient values and carbon footprint obtained as the average of the corresponding values of the following fruits: clementine, mandarin, orange, pomelo, tangerine, apple, apricot, avocado, banana, cherry, fig, grapes, kiwi, melon, peach, pear, pineapple, plum, watermelon, and strawberry.

^2^The ranges were obtained from average and standard deviation of the RDAs (18) for pupils from six to eleven years old.

^3^The population reference daily intake of vitamin B_12_ for the considered age group is equal to 1.6 μg. Apart from the 1 μg provided by breakfast, it is assumed that lunch and dinner have to provide 0.35 μg and 0.25 μg, respectively.

^4^This is due to the daily and weekly nature of the constraints.

^5^This happens when different recipes have the same ingredients with the same quantity, i.e., they have only a different food preparation such as, for example, Backed potatoes, Boiled potatoes with olive oil, Crispy backed potatoes, and Sauteed potatoes (see Supplementary Table SM1).

^6^The constraints assigned on the frequencies of dairy, eggs, fish, white and red meat, determine that there is exactly one dish for every food group each week. The variety of menu can be improved with more sophisticated constraints for a menu covering more weeks.

^7^To be more precise, the menu is a little bit richer in protein, that is within 19 and 44 g per day, and 144 and 185 g per week. Sodium intake is instead quite high and this is mainly due to dishes with a very high content of salt, such as quiche Lorraine and pizza.

^8^The EU's ambitious target is to reduce net emissions by at least 55% by 2030 compared to 1990 and become the first climate-neutral continent by 2050 (30). Since 1990 EU emissions have already dropped by approximately 26.4% in 2019 (31), a 55% reduction target, using 1990 as a baseline, represents about a 40% reduction target using 2019 (or today's) emissions as a baseline.

## References

[1] Crippa M Solazzo E Guizzardi D Monforti-Ferrario F Tubiello FN Leip A. Food systems are responsible for a third of global anthropogenic GHG emissions. Nat Food. (2021) 2:198–209. 10.1038/s43016-021-00225-9 37117443

[2] Communication from the Commission to the European Parliament the European Council the Council the the European economic social Committee the Committee of the Regions. The European Green Deal, COM/2019/640 final, Brussels. (2019). Available online at: https://eur-lex.europa.eu/legal-content/EN/TXT/?uri=COM:2019:640:FIN (accessed July 22, 2022).

[3] Farm, to Fork Strategy. For a Fair, healthy and Environmentally-Friendly Food System. Available online at: https://ec.europa.eu/food/system/files/2020-05/f2f_action-plan_2020_strategy-info_en.pdf (accessed July 22, 2022).

[4] Sustainable diets and biodiversity: directions and solutions for policy research and action. In: Proceedings of the International Scientific Symposium Biodiversity and sustainable diets united against hunger, FAO Headquarters. Rome (2010).

[5] Belgacem W Mattas K Arampatzis G Baourakis G. Changing dietary behavior for better biodiversity preservation: a preliminary study. Nutrients. (2021) 13:2076. 10.3390/nu13062076 34204478 PMC8234216

[6] Nelson ME Hamm MW Hu FB Abrams SA Griffin TS. Alignment of healthy dietary patterns and environmental sustainability: a systematic review. Adv Nutr. (2016) 7:1005–25. 10.3945/an.116.012567 28140320 PMC5105037

[7] McGuire S. Scientific Report of the 2015 Dietary Guidelines Advisory Committee. Washington, DC: US Departments of Agriculture and Health and Human Services. Adv Nutr. (2016) 7:202–4. 10.3945/an.115.011684 26773024 PMC4717899

[8] Rabès A Seconda L Langevin B Allès B Touvier M Hercberg S. Greenhouse gas emissions, energy demand and land use associated with omnivorous, pesco-vegetarian, vegetarian, and vegan diets accounting for farming practices. Sustain Prod Consum. (2020) 22:138–46. 10.1016/j.spc.2020.02.010

[9] Fjellstrom C. Mealtime and meal patterns from a cultural perspective. Scand J Nutr. (2004) 48:161–4. 10.1080/11026480410000986

[10] Eustachio Colombo P Patterson E Lindroos AK Parlesak A Schäfer Elinder L. Sustainable and acceptable school meals through optimization analysis: An intervention study. Nutr J. (2020) 19:61. 10.1186/s12937-020-00579-z 32580743 PMC7315552

[11] Ribal J Fenollosa ML García-Segovia P Clemente G Escobar N Sanjuán N. Designing healthy, climate friendly and affordable school lunches. Int J Life Cycle Assess. (2016) 21:631–45. 10.1007/s11367-015-0905-8

[12] Aliyar R Gelli A Hamdani SH. A review of nutritional guidelines and menu compositions for school feeding programs in 12 countries. Front Public Health. (2015) 3:148. 10.3389/fpubh.2015.00148 26301209 PMC4524891

[13] Ministero Della Salute. Dipartimento per la Sanità Pubblica Veterinaria, la Nutrizione e la Sicurezza Degli Alimenti. Direzione Generale Della Sicurezza Degli Alimenti e Della Nutrizione, “Linee di indirizzo nazionale per la ristorazione scolastica”. Conferenza Unificata. Provvedimento 29 aprile 2010. Intesa, ai Sensi Dell'art.8, Comma 6, Della Legge 5 Giugno. (2003). n.131, G.U. n. 134 del 11-6-2010. Available online at: https://www.salute.gov.it/imgs/C_17_pubblicazioni_1248_allegato.pdf (accessed July 22, 2022).

[14] Ministero della Salute. Direzione Generale per L'igiene e la Sicurezza Degli Alimenti e Della Nutrizione—Uff. 5–Nutrizione e Informazione ai Consumatori, Linee di indirizzo rivolte agli enti gestori di mense scolastiche, aziendali, ospedaliere, sociali e di comunità, al fine di prevenire e ridurre lo spreco connesso alla somministrazione degli alimenti; Ministero della Salute, Rome, Italy. (2018). Available online at: http://www.salute.gov.it/imgs/C_17_pubblicazioni_2748_allegato.pdf (accessed July 22, 2022).

[15] Benvenuti L De Santis A. Making a sustainable diet acceptable: an emerging programming model with applications to schools and nursing homes menus. Front Nutr. (2020) 7:562833. 10.3389/fnut.2020.562833 33240916 PMC7677360

[16] Benvenuti L De Santis A Santesarti F Tocca L. An optimal plan for food consumption with minimal environmental impact: the case of school lunch menus. J Clean Prod. (2016) 129:704–13, 10.1016/j.jclepro.2016.03.051

[17] Rossi L Ferrari M Martone D Benvenuti L De Santis A. The promotions of sustainable lunch meals in school feeding programs: the case of Italy. Nutrients. (2021) 13:1571. 10.3390/nu13051571 34067077 PMC8151658

[18] Società Italiana di Nutrizione Umana (SINU). Livelli di Assunzione di Riferimento di Nutrienti ed Energia per la Popolazione Italiana (LARN), 4th ed.; Istituto Nazionale di Ricerca per gli Alimenti e la Nutrizione (INRAN), attualmente Centro di Ricerca per gli Alimenti e la Nutrizione. SICS, Roma, Italy, (2014).

[19] CREA Centro di Ricerca Alimenti e Nutrizione. Available online at: https://www.alimentinutrizione.it/tabelle-nutrizionali/ricerca-per-alimento

[20] Ciqual French Food Composition Table. (2020). Available online at: https://ciqual.anses.fr/# (accessed July 22, 2022).

[21] Petersson T Secondi L Magnani A Antonelli M Dembska K Valentini R. A multilevel carbon and water footprint dataset of food commodities. Sci Data. (2021) 8:127. 10.1038/s41597-021-00909-8 33963181 PMC8105407

[22] Kolbe K. Mitigating climate change through diet choice: Costs and CO2 emissions of different cookery book-based dietary options in Germany. Adv Clim Change Res. (2020) 11:392–400. 10.1016/j.accre.2020.11.003

[23] Centro di Ricerca Alimenti e Nutrizione del Consiglio per la Ricerca in Agricoltura e l'analisi dell'economia Agraria (CREA). Linee Guida per Una Sana Alimentazione. Revisione 2018, CREA, Rome, Italy. (2019).

[24] Martone D Roccaldo R Censi L Toti E Catasta G D'Addesa D. ZOOM8 Study Group, Food consumption and nutrient intake in Italian school children: Results of the ZOOM8 study. Int J Food Sci Nutr. (2013) 64:700–5. 10.3109/09637486.2013.775226 23480239

[25] Sette S Le Donne C Piccinelli R Arcella D Turrini A Leclercq C. 2005-6 Study Group, The third Italian national food consumption survey, INRAN-SCAI 2005-06–part 1: nutrient intakes in Italy. Nutr Metabol Cardiovasc Dis. (2011) 21:922–32. 10.1016/j.numecd.2010.03.001 20674305

[26] WHO. Guideline: Sodium Intake for Adults and Children. World Health Organization, Geneva, Swiss, (2012). 23658998

[27] Società Italiana di Nutrizione Umana e Società Italiana di Scienze dell'alimentazione. Documento SINU –SISA per la Prima Colazione. Roma: SINU, SISA 2018 (in Italian).

[28] Red Red meat and processed meat/IARC Working Group on the Evaluation of Carcinogenic Risks to Humans IARC IARC monographs on the evaluation of carcinogenic risks to humans volume 114. IARC, Lyon, France, (2018).

[29] Battaglia Richi E Baumer B Conrad B Darioli R Schmid A Keller U. Health risks associated with meat consumption: a review of epidemiological studies. Int J Vitamin Nutr Res. (2015) 85:70–8. 10.1024/0300-9831/a000224 26780279

[30] Communication from the Commission to the European Parliament the Council the the European economic social Committee the Committee of the Regions. Fit for 55%: delivering the EU's 2030 Climate Target on the way to climate neutrality, COM/2021/550 final, Brussels. (2021). Available online at: https://eur-lex.europa.eu/legal-content/EN/TXT/?uri=CELEX:52021DC0550 (accessed July 22, 2022).

[31] European Union emission inventory report 1990-2019. European Environment Agency, Report No. 05/2021. (2021). Available online at: https://www.eea.europa.eu/publications/lrtap-1990-2019/file (accessed July 22, 2022).

[32] Scarborough P Appleby PN Mizdrak A Briggs ADM Travis RC Bradbury KE. Dietary greenhouse gas emissions of meat-eaters, fish-eaters, vegetarians and vegans in the UK. Clim Change. (2014) 125:179–92. 10.1007/s10584-014-1169-1 25834298 PMC4372775

[33] Rosi A Mena P Pellegrini N Turroni S Neviani E Ferrocino I. Environmental impact of omnivorous, ovo-lacto-vegetarian, and vegan diet. Sci Rep. (2017) 7:6105. 10.1038/s41598-017-06466-8 28733610 PMC5522483

[34] Tyszler M Kramer G Blonk H. Just eating healthier is not enough: studying the environmental impact of different diet scenarios for Dutch women (31-50 years old) by linear programming. Int J Life Cycle Assess. (2016) 21:701–9. 10.1007/s11367-015-0981-9

[35] Eustachio Colombo P Elinder LS Lindroos AK Parlesak A. Designing nutritionally adequate and climate-friendly diets for omnivorous, pescatarian, vegetarian and vegan adolescents in Sweden using linear optimization. Nutrients. (2021) 13:2507. 10.3390/nu13082507 34444667 PMC8398609

[36] Chai BC van der Voort JR Grofelnik K Eliasdottir HG Klöss I Perez-Cueto FJA. Which diet has the least environmental impact on our planet? A systematic review of vegan, vegetarian and omnivorous diets. Sustainability. (2019) 11:4110. 10.3390/su11154110

[37] Rosi A Pellegrini N Lazzi C Neviani E Ferrocino I Di Cagno R. Comparison of the environmental impact of omnivorous, ovo-lacto-vegetarian, and vegan diet. Ann Nutri Metabol. (2015) 67(S1):530. 10.1159/000440895

